# Secondary analysis of influenza a virus-infected cells at single-cell resolution reveals host BANF1 response to individual and combinations of detected segments

**DOI:** 10.1186/s12985-026-03140-2

**Published:** 2026-03-20

**Authors:** Zach Fears, Bradford K. Berges, Miglena Manandhar, Gene S. Tan, Brett E. Pickett

**Affiliations:** 1 Department of Microbiology and Molecular Biology, Brigham Young University, Provo, UT USA; 2 Infectious Diseases, J. Craig Venter Institute, La Jolla, CA USA; 3 Division of Infectious Diseases, Department of Medicine, University of California San Diego, La Jolla, CA USA; 4 Herbert Wertheim School of Public Health and Human Longevity Sciences, University of California San Diego, La Jolla, CA USA

**Keywords:** Influenza A virus, Signaling pathways, Virus segments, Transcriptomics, Bulk RNA-sequencing

## Abstract

Influenza A virus (IAV) infections play a substantial role in human disease, hospitalization, and economic loss. In an effort to better understand the intracellular effects that IAV replication has on individual host cells, this study set out to analyze an existing single-cell RNA sequencing (scRNAseq) dataset to identify the different metabolic pathways disrupted in cells infected with IAV. Specifically, these analyses consisted of calculating differential expression, signaling pathway enrichment, and text mining methods on a publicly available dataset consisting of 2041 mammalian MDCK cells infected with H9N2 IAV to model cross-species spread of endemic avian influenza. Interestingly, this dataset detected cells that were infected by viruses that carried less than a full set of genome segments, allowing us to ascribe impacts on host gene expression to specific viral genes. The results from this analysis enabled us to observe (1) the differences in host cellular gene expression caused by individual IAV segments, (2) the effects of various segment combinations on the host response, and (3) the segment-specific disruptions in signaling pathways related to three categories: virus replication, host immune response, and cell cycle. Deeper examination of these trends will improve our understanding of the mechanistic effects of responses caused by IAV at the molecular level and improve the ongoing development of host-based anti-viral therapeutics.

**Supplementary Information:**

The online version contains supplementary material available at 10.1186/s12985-026-03140-2.

## Introduction

Influenza A virus (IAV) is a negative-sense segmented RNA virus that continues to pose a major global health threat due to its high mutation rate, antigenic shift, and ability to evade host immunity. Virions require eight distinct genome segments, which encode up to 18 proteins, to be replication competent. IAV infection results in a complex and dynamic interplay between viral replication and the host intracellular, extracellular, and immune responses [1–9]. This interplay contributes to the acute respiratory symptoms that typically accompany infection including fever, cough, sore throat, muscle aches, and fatigue. These signs and symptoms can lead to severe complications including pneumonia and acute respiratory distress syndrome, and death–particularly in vulnerable populations [10–12].

The replication cycle of Influenza A virus (IAV) is a complex, multistep process that takes place in both the cytoplasm and nucleus of the host cell. It begins with the binding of the viral hemagglutinin (HA) protein to sialic acid residues primarily on the surface of host respiratory epithelial cells, which facilitates viral attachment and entry via endocytosis [13, 14]. The acidic environment of the endosome enables the viral envelope to fuse with the endosomal membrane and release the viral ribonucleoproteins (vRNPs) into the cytoplasm [14]. These vRNPs are then transported into the nucleus, where viral genome replication and transcription occur. Specifically, the viral RNA-dependent RNA polymerase synthesizes messenger RNAs (mRNAs) [13, 15–17], while complementary RNA (cRNA) intermediates serve as templates for new viral genomes (vRNA) [15, 16]. The viral mRNAs are exported to the cytoplasm and translated into viral proteins. Structural proteins and newly synthesized vRNPs assemble at the plasma membrane, where budding occurs to form new virions [17–19], aided by the cleavage of host sialic acid by viral neuraminidase (NA), enabling virion release and infection of neighboring cells [17, 18].

Multiple previous studies have characterized the host transcriptional response to IAV infection [20–23]. In addition, multiple single-cell RNA-sequencing (scRNA-seq) studies have revealed that not all infected cells receive or express all eight viral segments, resulting in a heterogeneous landscape of segment combinations within individual cells [24–27]. This phenomenon, often referred to as incomplete or abortive infection, may lead to altered or incomplete viral replication and can influence the host cell fate and the immune responses [28–30].

Some in-depth analyses of the association of the heterogeneity of viral genome segment with host gene expression have been performed previously [23, 26–28, 31, 32]. However, these studies characterized viral transcription, innate immunity, and the host antiviral response to various H1N1 or H3N2 strains. As such, the aim of the current study is to perform a secondary analysis of a prior scRNA-seq dataset to better characterize the host intracellular transcriptional response to the presence of various combinations of H9N2 IAV segments. The findings of this study could then be used to augment our understanding of host-pathogen interactions that could lead to novel therapeutic strategies in more human-relevant viral subtypes.

## Methods

### Dataset description

Read count data from a publicly available single-cell RNA-sequencing (scRNA-seq) experiment (NCBI BioProject PRJNA559274) were retrieved from a prior study that focused on transcripts during the first round of replication [24]. This study was comprised of Madin-Darby canine kidney (MDCK) cells infected with H9N2 influenza A virus strain A/guinea fowl/Hong Kong/WF10/99 (GFHK99) for eight hours at a multiplicity of infection (MOI) values of 0.067, 0.2, 0.6, and 1.8 [33]. These scRNA-seq read counts were then analyzed following an established analytical workflow using the R Seurat package [34].

### Preprocessing and quality control

To begin, the pre-computed read counts were imported into R according to their multiplicity of infection (MOI) prior to merging them into a single table. Merging was done under the assumption that MOI reflects a Poisson distribution that represents the probability that any given cell in the population is infected, and that MOI is independent of any intracellular host response after infection. Quality control metrics were then used to filter out cells that did not meet the acceptable minimum criteria including unique molecular identifier (UMI) counts per cell (> 500), genes per cell (> 300), log_10_ genes per UMI (> 0.725), and mitochondrial counts ratio (< 0.2). A cutoff value of 0.725 was used for genes per UMI since there was minimal risk of low complexity cell contamination due to two reasons (1) the MDCK cell line is expected to have relatively low complexity, and (2) the samples derived from this cell line were generally expected to be homogenous. After filtering the results with these threshold criteria, cells were grouped according to which combinations of IAV genomic segments were detectable in each cell. This approach generated 24 unique combinations of segments among infected cells as well as a subset of cells in which no influenza segments were detected (hereafter referred to as “clean group”, with the latter being used as the control for downstream differential comparisons. Group IDs representing each unique combination were added to the metadata for each group of cells prior to combining all IAV groups with the clean group into one Seurat object. These read counts were then normalized using the NormalizeData function in Seurat.

### Differential expression analysis

Eight subpopulations of infected cells were then selected for further analysis based on both the relative abundance of cells and the number of affected genes in those cells. The FindMarkers function in Seurat was used to determine the differentially expressed genes (DEGs) for each of the individual infected IAV groups as compared to the clean group to calculate the DEGs. Each set of DEGs was then filtered by whether they had a |0.6| average log_2_ fold-change and adjusted p-values < 0.05. Human orthologs for each canine gene in the MDCK cells were then retrieved using the homologene R package.

### Pathway enrichment

These human orthologs were then used as input to the R enrichR package for pathway enrichment [35], maintaining only those pathways that were statistically significant (FDR-adjusted p-value < 0.05). The list of DEGs for each group was then further filtered, keeping only those that had human homologs.

### Text-mining procedures

Then, using the tm (text mining) R package, the lists of enrichR outputs for each IAV group DGE profile were converted to a text mining corpus. All terms were cleaned to ensure consistency by formatting all terms to lowercase and removing their suffixes. Then common “stopper” words were removed (e.g. ‘the’, ‘and’, and ‘of’) as well as commonly repeated scientific terms (e.g. “complex”, “human”, and “disease”). The remaining corpus of words was sorted to produce counts for words that are known to be related to (1) cell cycle, (2) viral and antiviral responses, and (3) general immune responses. Each of these categories represents how many cell cycle, viral, and immune-related pathways were present in the enrichR output generated from the DGE profile for each IAV group. The counts for each word group were then aggregated using the tidyverse R package and plotted using ggplot2.A new list was then constructed for each gene across all significant enrichR pathways according to group, representing pathways stratified by IAV group and gene. The same text processing procedure as above was applied to each of these sub-enrichR outputs to produce a count of cell cycle, viral/antiviral, and general immune pathways by gene in the IAV group DGE profile. Counts were then aggregated using tidyverse and plotted using ggplot2.

## Results

We began by applying various quality control metrics to remove low-quality cells. from the prior scRNAseq dataset. Once this was complete, we categorized the cells based on the one (or more) unique combinations of IAV segment(s) that were detected in each cell (Table 1). This approach enabled us to identify 24 subpopulations of infected cells (Supplemental Table 1), each comprised of a unique combination of detectable segments, and one additional group of cells with no detectable presence of any IAV segment. We then compared the host gene expression profile from each infected group to the uninfected group and applied various filtering criteria to the results from this analysis to maximize the detection of DEGs. Specifically, the filtering criteria for this step included > ± 0.6 log_2_ fold-change values and FDR-adjusted p-value < 0.05. Of the 26 total host DEGs among the different groups, we identified15 host genes that had not been previously implicated in IAV infection (Table 2). Of these 15 genes, only one, COX8A, was differentially expressed when segments encoding hemagglutinin, nucleoprotein, neuraminidase, matrix proteins, and nuclear export protein (segments 4, 5, 6, 7, and 8 respectively) were detected in the cell, making it a previously unknown host-pathogen interaction that was only discoverable single cell resolution. ISG15, an interferon-activated ubiquitin-like protein, was also found to be upregulated only when segments 4,5,6,7, and 8 were present.

**Table 1:** Segment numbers, canonical gene product abbreviations and descriptions of gene products produced by segments encapsidated in IAV

IAV segment number	Abbreviation	Encoded gene products
1	PB2	Polymerase Basic 2, viral polymerase subunit
2	PB1	Polymerase Basic 1, viral polymerase subunit
3	PA	Polymerase Acid, viral polymerase subunit
4	HA	Hemagglutinin
5	NP	Nucleoprotein, forms viral ribonucleoprotein complexes
6	NA	Neuraminidase
7	M	Matrix proteins M1 and M2
8	NS	Non-structural proteins NS1 and NS2/NEP

**Table 2:** List of Host Genes Not Previously Implicated in IAV Infection Found from a DGE Analysis Comparing Infected Cells with Unique Combinations of Detected IAV Segments vs. Cells with No Detectable IAV Segments

Gene symbol	Gene name	Detected segments*	Log_2_FC	Adj. *p*-value
COX8A	Cytochrome c oxidase subunit 8 A	45,678	−0.6106681	2.22E-02
TRAPPC5	trafficking protein particle complex subunit 5	12,345,678	0.7610334	1.55E-36
RUSC1	RUN and SH3 domain containing 1	12,345,678	0.7209379	2.47E-36
CILP2	Cartilage intermediate layer protein 2	12,345,678	0.6565764	6.69E-32
RPL27A	Ribosomal protein L27a	12,345,678	0.6578284	2.45E-30
GJC2	Gap junction protein gamma 2	12,345,678	0.6792911	8.62E-29
RPS21	Ribosomal protein s21	12,345,678	−0.6359383	2.41E-25
MTHFD2	Methylenetetrahydrofolate dehydrogenase (NADP+ dependent) 2, methenyltetrahydrofolate cyclohydrolase	12,345,678	0.6464787	1.56E-23
MDH1	Malate dehydrogenase 1	12,345,678	0.6261138	2.91E-23
FDPS	Farnesyl diphosphate synthase	12,345,678	0.6343338	2.62E-21
ANKRD11	Ankyrin repeat domain containing 11	12,345,678	0.6130471	6.55E-21
LGALS1	Galectin 1	12,345,678	0.6601219	1.05E-19
UBB	Ubiquitin B	12,345,678	0.6687978	5.31E-18
METAP2	Methionyl aminopeptidase 2	12,345,678	−0.6055476	3.92E-14
TMA16	Translation machinery associated 16 homolog	12,345,678	−0.6131601	5.21E-12

*Each digit represents the IAV segment numbers that were detected in cells with differential expression of the indicated host gene

We also observed seven DEGs that were dysregulated across multiple combinations of detectable IAV segments. As such, we wanted to determine whether the direction of this change (significantly upregulated or downregulated) was consistent across the relevant segment combinations. We consequently compared the log_2_ fold-change values for these genes across the different segment combinations (Table 3; Supplemental Table 2). Overall, the results from this analysis suggest that as the diversity of detectable IAV segments increases, the host transcriptional response becomes more complex. Interestingly, although the magnitude of differential expression across this set of genes varied, the directionality for any given gene was consistent regardless of the combinations of segment(s) that were detected in the groups of cells.

**Table 3:** List of DEGs with Highest Log_2_ Fold-Change Values by Combination of IAV Genome Segments Detected

Gene symbol	Gene name	Log_2_FC 8*	Log_2_FC 5*	Log_2_FC 2,345,678*	Log_2_FC 12,345,678*
BANF1	Barrier to autointegration nuclear assembly factor 1	0.6502034	0.8847999	1.088515	1.371576
ISG20	Interferon stimulated exonuclease gene 20		−0.7327589		−0.7374079
DDX58	RNA sensor RIG-I		−0.796534		−1.002196
MX2	MX dynamin like GTPase 2		−0.850723		−1.067351
ND4L	NADH dehydrogenase subunit 4 L			−0.8511553	−1.3631495
RPL21	Ribosomal protein L21			−0.7403023	−0.9381612
LPCAT4	Lysophosphatidylcholine acyltransferase 4			0.8613392	1.1245697

*Each digit in the column header represents the IAV segment numbers (1–8) that were detected in cells with differential expression of the indicated host gene. As such, each column showcases the log_2_FC measured between the subset of infected cells having detectable expression of the one (or more) designated IAV segment(s) vs. the mock-infected cells

**Fig. 1:**
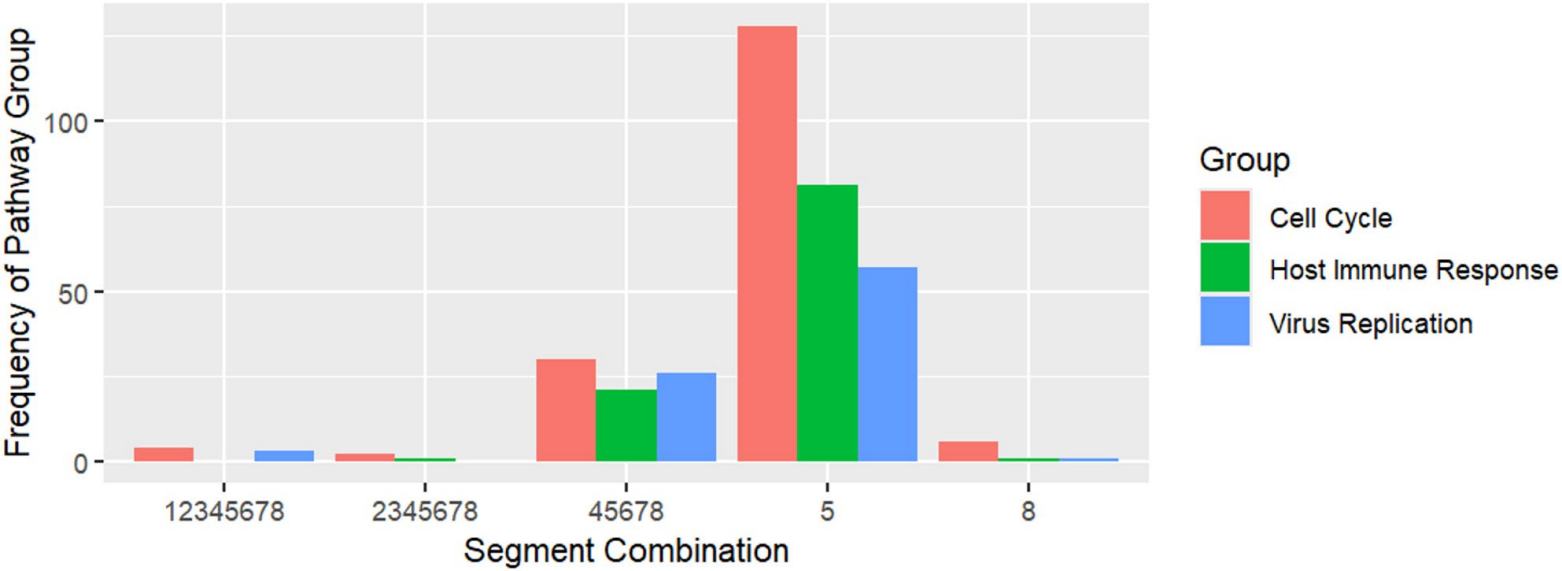
Barchart Representing the Raw Frequency (Counts) Across the Three Pathway Groups Affected by the Presence of Various Detected IAV Genome Segment Combinations. The numerical frequency of the three pathway groups among the five unique combinations of IAV genomic segments that were detected in the subpopulations of host cells. Pathway groups were generated from DEGs calculated from infected with designated segments detected vs. mock-infected cells. Effects of IAV segment combinations on the host cell were color-coded by processes including cell cycle (red), host response to viral infection (blue), or host immune response (green)

We next wanted to determine whether there were any host intracellular signaling pathways that were significantly affected by each of the 24 combinations of viral genome segments. To do so, we took the DEG list from each of the segment combinations and performed pathway enrichment analysis using the enrichR package. We then applied text mining tools to group the pathway results based on their relevance/association to cell cycle, viral/antiviral responses, or any other general immune responses (Fig. 1). Interestingly, we found that more than 100 cell cycle-related pathways were affected when only the segment encoding a nucleoprotein (segment 5) was detected in the cell. We also observed differences in the proportions of cell cycle-related pathways to viral pathways across the different segment combinations.

**Fig. 2:**
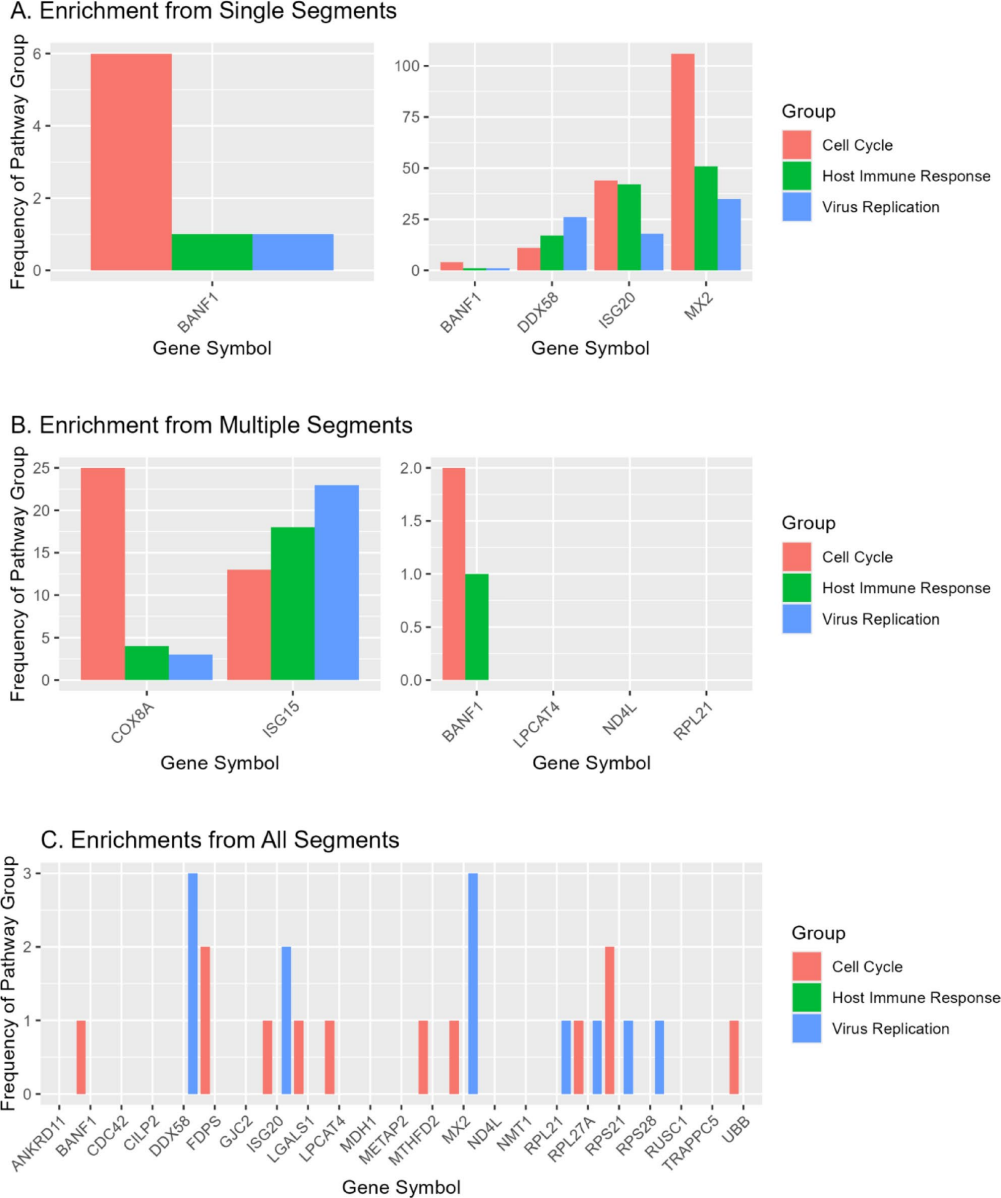
Comparing Disrupted Pathways in Selected Categories Between Cells with All IAV Segments and Cells with Any Number of IAV Segments. Bar graphs plotting the raw frequency counts of the pathway groups among the DEGs in each significant flu comparison group. Each panel takes one of the comparison groups from Fig. 1 and counts how many of those pathways each DEG is a member of. (**A**) Pathway groups for IAV segments eight (left panel) and five (right panel). (**B**) Pathway groups from combinations of segments 45,678 (left panel) and 2,345,678 (right panel). (**C**) Pathway groups across all segments. The x-axis for each graph is labeled with each DEG found from the comparison of each respective infection group. Pathway groups were formed from enrichR pathways involving cell cycle processes (red), response to viral infection (blue), or host immune response (green). Certain DEGs were implicated in more cell cycle-related pathways than the rest of the DEGs of the same comparison, including COX8A, MX2, and BANF1. Some DEGs were not implicated in any cell-cycle, host immune response, or virus replication pathways, and therefore show no bars above them

We next wanted to determine which genes were most strongly associated with the categories of pathways (i.e. cell cycle, immune, and viral). We consequently examined the individual DEG-affected pathway groups and separated them by which DEGs were present. We then applied a similar text mining grouping technique as was used previously to produce groups of cell cycle, viral, and immune pathways for each DEG in the IAV segment combination group. This analysis revealed pathways such as those characterized and implicated in B-cell lymphomas (cell cycle), Ebola virus disease (viral), and Immune deficiency familial variable (immune) (Fig. 2). We found that MX2, COX8A, BANF1 were major contributors to the number of cell cycle-related pathways in cells that only included segment combination 5, 45,678, and 2,345,678 respectively. The consistent presence of the nucleoprotein (segment 5) across these groups was somewhat unexpected and suggests a possible higher-level role in regulating the host cell cycle.

Given the observed impact of the seven host factors listed in Table 3 on multiple intracellular processes, we reviewed publicly available IAV-focused bulk RNA-sequencing studies to determine whether our findings in a canine cell line were relevant to infected human cell lines (Table 4). Specifically, we examined results from a prior study that evaluated differential gene expression in four human cell lines infected with a A/Puerto Rico/8/1934 isolate after 48 hrs [36]. That study identified ISG20, DDX58, and MX2 as being significantly upregulated in WI-38 VA-13 and MRC-5 lung fibroblast cells as well as in A549 alveolar basal epithelial cells. That study also reported RPL21 to be upregulated in both HEK293FT kidney cells and in A549 cells; while LPCAT4 was upregulated only in HEK293FT cells.

**Table 4:** Log_2_ fold-change values and multiple hypothesis adjusted p-values for host factors that were reported in a public study that infected multiple human cell lines for 48 h with the A/Puerto Rico/8/1934 isolate, which overlapped with the seven key host factors in the present study

	A/Puerto Rico/8/1934 (H1N1)							
	WI-38 VA-13 cells (48hpi vs. mock-infected)		MRC-5 cells (48hpi vs. mock-infected)		A549 cells (48hpi vs. mock-infected)		HEK293FT cells (48hpi vs. mock-infected)	
Gene Symbol	log2FC	padj	log2FC	padj	log2FC	padj	log2FC	padj
BANF1	NR	NR	NR	NR	NR	NR	NR	NR
ISG20	3.6	0	3.2	0	2	0	NR	NR
DDX58	3.2	0	2.3	0	3.9	0	NR	NR
MX2	5.6	0	3.4	0	7.1	0	NR	NR
ND4L	NR	NR	NR	NR	NR	NR	NR	NR
RPL21	NR	NR	NR	NR	1.5	0	1.1	0
LPCAT4	NR	NR	NR	NR	NR	NR	1.2	0

log2FC: log2 fold-change

padj: multiple-hypothesis adjusted p-value

NR: Not Reported

We next wanted to determine whether our host factor results in a H9N2 isolate were relevant to human-tropic subtypes by comparing them to the results from a separate public study that generated bulk RNA-sequencing data from human bronchial epithelial BEAS-2B cells infected with one of three H3N2 isolates: BR10/07, PER16/09, or UD/72 after a maximum of 24 hrs [37] (Table 5). The public data from showed that three of our seven host factors of interest DDX58, MX2, and RPL21 were significantly upregulated in a cell line infected with H3N2 viruses and at least partially support the observations made in this work.

**Table 5:** Log_2_ ratio values and multiple hypothesis adjusted p-values for host factors that were reported in a public study that infected human BEAS-2B cells for up to 24 h with a BR10/07, PER16/09, or UD/72 isolate; which overlapped with the seven key host factors identified in the present study (bold numbers indicate the highest log_2_ ratio values in each row)

	BEAS-2B cells					
	log2ratio 24hpi vs. mock A/Brisbane/10/2007 (H3N2)		log2ratio 24hpi vs. mock A/Perth/16/2009 (H3N2)		log2ratio 24hpi vs. mock Udorn/307/72 (H3N2)	
Gene symbol	log_2_ ratio	padj	log_2_ ratio	padj	log_2_ Ratio	padj
BANF1	NR	NR	NR	NR	NR	NR
ISG20	NR	NR	NR	NR	NR	NR
DDX58	1.774644256	4.22E-21	2.957988501	1.75E-48	**3.60852194**	9.91E-41
MX2	1.582296252	5.49E-36	**4.159011841**	1.49E-98	3.77551508	2.01E-70
ND4L	NR	NR	NR	NR	NR	NR
RPL21	0.729655981	2.31E-03	0.944231689	7.99E-05	**1.50985324**	3.38E-07
LPCAT4	NR	NR	NR	NR	NR	NR

Bold represents the largest log ratio in each row

NR: Not Reported

## Discussion

The current study reports the results of our secondary analysis of an existing scRNAseq dataset derived from infecting MDCK cells with different MOIs (0.067, 0.2, 0.6, and 1.8) of the GHK99 (H9N2) isolate of IAV, which served as a model for endemic avian influenza. Our approach to stratify the infected cells into 24 distinct segment-defined groups enabled us to detect fine-grained patterns of host gene dysregulation that would be masked in bulk or segment-agnostic analyses. This approach led to the identification of 26 host DEGs, 15 of which are novel associations with IAV infection. These results emphasize the power of our segment-specific strategy for predicting novel host-pathogen interactions. Interestingly, we found seven host genes that were dysregulated across more than one combination of IAV segments. We then applied text-mining tools to assign the different intracellular signaling pathways into one of three groups: (1) cell cycle, (2) host response to viral infection, and (3) host immune response. Subsequent analyses identified a subset of host genes that appeared to play a role in at least one pathway category.

Despite the diversity of segment combinations that were detected, the direction of the differential expression (positive or negative) for each gene remained surprisingly consistent. This suggests either a robust and potentially consistent host response once a threshold of viral genetic material is present, or potentially that a portion of the transcriptional response relies on specific driving host factors. Moreover, we observed a trend of increasing host gene differential expression in parallel with increasing segment count, which may reflect a dose-dependent or combinatorial effect of the viral gene products on the host transcriptional machinery. Among the DEGs, three may be of practical significance given their statistical significance across multiple viral segment combinations and across multiple signaling pathways. These three DEGs include Barrier To Autointegration Nuclear Assembly Factor 1 (BANF1; BAF), MX Dynamin Like GTPase 2 (MX2), and the mitochondrial inner-membrane Cytochrome C Oxidase Subunit 8 A (COX8A).

Upregulation of BANF1 was especially of interest as it was identified after analyzing all groups of segment combinations, increasing in upregulation as the number of detected IAV segments increased. This observation suggests that the magnitude and variety of statistically significant up- or downregulation observed in at least a subset of the host transcriptome increases as more IAV segment combinations are detected. BANF1 is a non-specific DNA-binding protein that has been associated with infection with influenza A virus[38–40], as well as other human-tropic viruses including Human Immunodeficiency Virus [41, 42], Human Papillomavirus [43], and Vaccinia virus [38, 44]. Specifically, BANF1 has previously been shown to be an upstream effector of cyclic GMP-AMP synthase (cGAS) and to the stimulator of interferon genes signaling (STING) protein, which contribute to the innate immune response [45, 46]. Specifically, STING and cGAS affect expression of interferon-stimulated genes that is independent from type-I interferon and Signal Transducer and Activator of Transcription 1 (STAT1) signaling cascades [47]. The ubiquity of BANF1 upregulation across the different infection groups warrants further experiments to confirm whether STING and cGAS activity, as well as other host factor(s), are also dysregulated as more IAV segments are detected.

When analyzing significantly affected pathways, we found that the host MX2 gene product likely plays a large role in the cell cycle-related pathways in cells having segment five (nucleoprotein; NP) detected. This, and that cell cycle pathways included the most dysregulated pathways of all analyzed groups, both in relation to cell cycle and overall is likely worthy of additional experiments. MX2 is an interferon-activated GTPase implicated in antiviral responses especially during retrovirus infection [48, 49], and recently during influenza A virus infection [20]. The antiviral role of MX2 is dependent on its oligomerization state, binding to viral components, and interactions with other host proteins [49, 50].

COX8A is the terminal component of the mitochondrial electron transport chain and therefore has an important role in ATP generation. This DEG was found to only be significantly downregulated when not all IAV genomic segments were present in the cell. COX8A has been identified as an important host factor that contributes to disease in pseudorabies virus and Epstein-Barr virus [51], but to our knowledge, has not been identified in IAV infection previously. Downregulation of COX8A would decrease the amount of ATP that is produced, which would affect various energy-driven anti-viral and cellular processes. Interestingly, prior experiments that inhibited COX-1 and COX-2 in mice showed increased mortality and decreased production of TNF-alpha [52]. While COX8A was only downregulated in an infection group lacking half of the flu segments, its differential expression suggests that the host response is at least partially dependent on the number of IAV segments that successfully enter and reproduce in the host cell.

Our pathway enrichment analysis uncovered a surprising number of cell cycle-related pathways that were affected in each infection group, often including only a consistent subset of the DEGs present in the majority of the pathways. These analyses further demonstrated the nuanced impact of segment-specific infections on host cell function. Notably, cells containing only segment five (nucleoprotein or NP) or combinations that include segment five displayed a disproportionately large number of dysregulated pathways associated with cell cycle control. The NP gene product has been shown to minimize the pathogen-associated molecular patterns within the viral RNA from being recognized by RIG-I, which would contribute to a lack of intracellular innate immunity activation [53–56].

Of the eight IAV segments, only segments five (NP) and eight (NS) are detected in nearly all of the comparison groups, while also being the only two of three segments detected in isolation from other IAV segments (segment 7 was detected in isolation as well but our analysis produced no significant DEGs). Comparing these two isolation comparison groups, the group with only segment five being detected has more DEGs and considerably more disrupted pathways than the group with only segment eight, causing our attention to be drawn to the role of segment five in infection. Interestingly, the IAV NP gene product has been reported to be present at higher levels in G0/G1-phase cells than in G2/M-phase cells [57]. As such, our findings suggest that IAV can dysregulate cell-cycle progression regardless of whether all, or even most, segments are present. NP oligomerization and its ability to bind RNA non-specifically could at least partially explain its role in cell cycle changes [58–61]. More specifically, IAV requires active transcription to provide host mRNAs for PB2 cap-binding activity, which is consistent with intracellular processes occurring during the G0/G1 phase. Characterizing the mechanisms of the NP segment in dysregulating cell cycle-related processes justifies continued experimentation. Given past studies, we do not believe that the presence/absence of individual segments strongly impacts the overall ability of IAV to infect and replicate in the host, given the presence of complementing segments during natural infection [30, 62–65].

Although the current study was performed in canine cells with a H9N2 virus, five of the seven gene products that we predicted to play a role in the host intracellular response had been reported in human cell lines infected with H1N1 or H3N2 viruses [36, 37]. Although we found BANF1 to be consistently differentially expressed across multiple combinations of segments, it has not been reported previously as playing such a direct role in IAV infection. In particular, cGAS can be inactivated when participating in binding interactions including a physical interaction with nucleosomes or with the barrier-to-autointegration factor 1 (BAF) protein [66, 67]. IAV infection can result in destruction of the mitochondria, which subsequently activates cGAS-STING signaling through the recognition of cytoplasmic mtDNA [68, 69]. Additional experiments will be needed to better characterize whether canonical or non-canonical roles for BANF1, ND4L, and COX8A are involved in the detection and response to mitochondrial damage and oxidative stress. Validation work that quantifies the extent to which the activity of these host factors depends on the presence of at least one IAV segment combinations, and/or on the viral subtype, would be of particular interest.

Though results from the current study are informative, some inherent limitations exist due to the scope of the original experiment, which was to better characterize the effect of defective particles and incomplete genome packaging in infected cells. As such, this scRNA-seq dataset yielded substantially fewer infected cells than is typically expected in single cell studies due to the relatively low MOI that was used to facilitate the differentiation of infected and uninfected cells. Consequently, we were limited in our analysis to studying the impact that a select few IAV segment combinations had on the host immune response as opposed to a more robust study on the wider array of combinations or even the abundance of specific IAV segments. Although this resulted in a relative dearth of data available for analysis on all possible infection groups, the number of cells in each group was robust enough to generate results that were both statistically significant and biologically relevant. Unfortunately, deconvoluting the contribution of multiple segment combinations in this cell line with this IAV subtype is not possible with the current dataset. We believe that future studies that use alternative experimental designs, detection technologies, and/or analytical approaches may better elucidate the expression patterns of the host response across a larger range of infection profiles. Additional future analyses could also evaluate any intracellular expression changes that were associated with the different MOIs. Such studies could also suggest mechanisms for the observed differences in DEGs between the various groups reported in this study and, potentially, augment our understanding of the contribution of unique combinations of IAV segments to the host response.

Together, our findings demonstrate that infection with different combinations of IAV genomic segments triggers somewhat distinct host transcriptional and pathway responses. This highlights the need to consider the segmental composition of IAV infection when interpreting host response data, especially in the context of defective particles and incomplete genome packaging. We believe that future work that quantitatively characterizes segment-specific host-pathogen interactions could inform therapeutic strategies targeting particular stages of the IAV life cycle or its manipulation of host processes.

## Supplementary Information

Supplementary Material 1: All combinations of IAV segments detected in individual cells present after initial quality control, the number of cells with that combination present, how many DEGs they had, how many of those DEGs had human orthologs, and how many pathways included those human ortholog DEGs

Supplementary Material 2: All host genes that were significantly differentially expressed in cells with detected influenza segments

## Data Availability

Not applicable.
